# Supplementary material for: The Effect of Conjugated Nitrile Structures as Acceptor Moieties on the Photovoltaic Properties of Dye-Sensitized Solar Cells: DFT and TD-DFT Investigation
Source: Int J Mol Sci. 2024 Jun 28;25(13):7138. doi: 10.3390/ijms25137138 (PMC11241837; doi:10.3390/ijms25137138)
Supplement: Supplementary file 1 [file ijms-25-07138-s001.zip › ijms-3065836-supplementary.pdf]

Table S1 online sources for these compounds for the selected Nitrile Structures

Compounds	Link
Cy-1	https://pubchem.ncbi.nlm.nih.gov/compound/5975
Cy-2a	https://pubchem.ncbi.nlm.nih.gov/compound/8010#section=3D-Conformer
Cy-2b	https://pubchem.ncbi.nlm.nih.gov/compound/637930#section=3D-Conformer
Cy-2c	https://pubchem.ncbi.nlm.nih.gov/compound/136705#section=3D-Conformer
Cy-3a	https://pubchem.ncbi.nlm.nih.gov/compound/139134#section=3D-Conformer
Cy-3b	https://pubchem.ncbi.nlm.nih.gov/compound/136784#section=3D-Conformer
Cy-4a	https://pubchem.ncbi.nlm.nih.gov/compound/300601#section=3D-Conformer
Cy-4b	https://pubchem.ncbi.nlm.nih.gov/compound/77282#section=3D-Conformer
Cy-4c	https://pubchem.ncbi.nlm.nih.gov/compound/1-Propene-1_1_3_3-tetracarbonitrile#section=3D-Conformer
Cy-4d	https://pubchem.ncbi.nlm.nih.gov/compound/54449303#section=3D-Conformer

Table S2 Chemical parameters for the same proposed design with known anchor substituents in the A-site.

Compounds	E_{HOMO} (eV)	E_{LUMO} (eV)	ΔE (eV)	IP (eV)	EA (eV)
cyanoacrylic acid	-5.519	-3.454	2.065	5.519	3.454
Phosphonic Acid	-5.390	-3.087	2.303	5.390	3.087
Sulfonic acid	-5.425	-3.059	2.366	5.425	3.059
Catechol Dye	-5.031	-2.548	2.483	5.031	2.548
Tetrazole Dye	-5.370	-3.156	2.214	5.370	3.156
Sulfonic acid	-5.425	-3.059	2.366	5.425	3.059
Carboxylic	-5.373	-2.956	2.417	5.373	2.956
